# *Loose Plant Architecture1* (*LPA1*) determines lamina joint bending by suppressing auxin signalling that interacts with C-22-hydroxylated and 6-deoxo brassinosteroids in rice

**DOI:** 10.1093/jxb/erw002

**Published:** 2016-01-29

**Authors:** Jing Miao Liu, Soon Ju Park, Jin Huang, Eun Jin Lee, Yuan Hu Xuan, Byoung Il Je, Vikranth Kumar, Ryza A. Priatama, Vimal Raj K, Sung Hoon Kim, Myung Ki Min, Jun Hyeon Cho, Tae Ho Kim, Anil Kumar Nalini Chandran, Ki Hong Jung, Suguru Takatsuto, Shozo Fujioka, Chang-deok Han

**Affiliations:** ^1^Division of Applied Life Science (BK21 Program), Plant Molecular Biology and Biotechnology Research Center (PMBBRC), Gyeongsang National University, Jinju 660-701, Republic of Korea; ^2^Department of Biological Science, Wonkwang University, Iksan, Jeonbuk 570-749, Republic of Korea; ^3^College of Plant Protection, Shengyang Agricultural University, Dongling Road 120, Shengyang, China110866; ^4^Department of Molecular Breeding Division, National Academy of Agricultural Science, RDA, Jeonju, Republic of Korea; ^5^Department of Southern Area Crop Science, NICS (National Institute of Crop Science), RDA, 20th Jeompiljaero, Miryang, Gyeongnam 627-803, Republic of Korea; ^6^National Academy of Agricultural Science, Rural Development Administration, Suwon, 441-857, Republic of Korea; ^7^Graduate School of Biotechnology & Crop Biotech Institute, Kyung Hee University, Yongin, 446-701, Republic of Korea; ^8^Department of Chemistry, Joetsu University of Education, Joetsu, Niigata 943-8512Japan; ^9^RIKEN Center for Sustainable Resource Science, Wako, Saitama 351-0198,Japan

**Keywords:** Auxin, C-22-hydroxylated BRs, lamina inclination, lamina joints, *Loose Plant Architecture1* (*LPA1*), *OsPIN*s, plant architecture, rice (*Oryza sativa*), 6-deoxos BRs.

## Abstract

Lamina inclination is a key agronomical character that determines plant architecture and is sensitive to auxin and brassinosteroids (BRs). *Loose Plant Architecture1* (*LPA1*) in rice (*Oryza sativa*) and its Arabidopsis homologues (*SGR5/AtIDD15*) have been reported to control plant architecture and auxin homeostasis. This study explores the role of *LPA1* in determining lamina inclination in rice. *LPA1* acts as a positive regulator to suppress lamina bending. Genetic and biochemical data indicate that *LPA1* suppresses the auxin signalling that interacts with C-22-hydroxylated and 6-deoxo BRs, which regulates lamina inclination independently of *OsBRI1*. Mutant *lpa1* plants are hypersensitive to indole-3-acetic acid (IAA) during the lamina inclination response, which is suppressed by the brassinazole (Brz) inhibitor of C-22 hydroxylase involved in BR synthesis. A strong synergic effect is detected between *lpa1* and *d2* (the defective mutant for catalysis of C-23-hydroxylated BRs) during IAA-mediated lamina inclination. No significant interaction between *LPA1* and *OsBRI1* was identified. The *lpa1* mutant is sensitive to C-22-hydroxylated and 6-deoxo BRs in the *d61-1* (rice *BRI1* mutant) background. We present evidence verifying that two independent pathways function via either BRs or *BRI1* to determine IAA-mediated lamina inclination in rice. RNA sequencing analysis and qRT-PCR indicate that *LPA1* influences the expression of three *OsPIN* genes (*OsPIN1a*, *OsPIN1c* and *OsPIN3a*), which suggests that auxin flux might be an important factor in *LPA1*-mediated lamina inclination in rice.

## Highlight

*LPA1* suppresses auxin signalling that interacts with C-22-hydroxylated and 6-deoxo brassinosteroids, which regulates lamina inclination independently of *OsBRI1*.

## Introduction

Rice (*Oryza sativa*) is a globally important cereal crop that provides carbohydrates for more than half of the world’s population. Planting density is a crucial factor for enhancing crop yields in a given area and the aerial plant structure or plant architecture affects crop yield. Rice leaf blades naturally bend downwards as plants become mature. Erect plant architecture is considered to be the ideal plant type and is continuously selected by farmers and breeders (Kovach *et al.*, 2007).

 Many genes have been reported to regulate leaf angle in rice and other plants. Rice *Leaf Inclination2* (*OsLC2*) is involved in leaf bending by influencing cell division and hormone-responsive genes (Zhao *et al.*, 2010). Rice *Leaf and Tiller Angle Increased Controller* (*OsLIC*) is an antagonistic factor of *BRASSINAZOLE-RESISTANT1* (*BZR1*) and has an epistatic relationship with BR synthesis and BR signalling mutants (Wang *et al.*, 2008; Zhang *et al.*, 2012). Rice *Increased Leaf Inclination1* (*OsILI1*) and its binding protein ILI1 Binding bHLH Protein1 (OsIBH1) function downstream of *BZR1* in BR-mediated regulation of cell elongation (Zhang *et al.*, 2009). *Increased Leaf Angle1* (*ILA1*) affects rice leaf angle by regulating mechanical tissue formation at the leaf lamina joint (Ning *et al.*, 2011). Wu *et al.* (2013) reported that *LPA1* encodes an INDETERMINATE DOMAIN protein regulating tiller and leaf angle. The Arabidopsis *OsLPA1* homologues (*AtIDD14*, *AtIDD15* and *AtIDD16*) control branch angles by regulating the expression of auxin biosynthetic and transport genes (Cui *et al.*, 2013).

 Early studies employing excised lamina joints revealed the effects of auxin and BRs on lamina inclination (Maeda, 1965). Exposure of lamina joints to exogenous auxin or BRs induces strong lamina inclination (Wada *et al.*, 1981). Such hormonal effects were later verified by genetic studies of mutants with altered lamina angles. *ILI1*, *Brassinosterioid Upregulated1* (*BU1*) and *OsLIC* encode transcription factors that determine lamina bending via BR synthesis or signalling pathways (Wang *et al.*, 2008; Zhang *et al.*, 2009). In fact, BR synthesis and signalling mutants exhibit erect phenotypes (Sakamoto *et al.*, 2006). Examination of rice *Leaf Inclination1* (*LC1*), encoding an IAA-amido synthetase (OsGH3-1), revealed the effects of auxin on leaf inclination (Zhao *et al.*, 2012), and the synergistic effects of BRs and auxin have been demonstrated (Takeno and Pharis, 1982; Mandava, 1988). A recent report proposed that there are two BR-mediated pathways that interact with auxin to regulate lamina joint bending: the C-22-hydroxylated and 6-deoxo BR-mediated pathway and an OsBRI1-mediated pathway (Nakamura *et al.*, 2009).

 Little information is available about the regulatory mechanisms that determine hormone-responsive lamina bending. In this study, we investigated the role of *LPA1* in IAA-mediated lamina bending. We demonstrate that a main function of *LPA1* in lamina inclination is to suppress the IAA signalling pathway that interacts with C-22-hydroxylated BRs, which is independent of *OsBRI1*. We provide genetic evidence verifying that two independent pathways, involving either BR compounds or the OsBRI1 receptor, function in IAA-mediated lamina inclination. In addition, RNA-seq analysis and qRT-PCR revealed that the expression levels of three *OsPIN* genes – *OsPIN1a*, *OsPIN1c* and *OsPIN3a* – are positively correlated with those of *LPA1*. This study increases our knowledge of the regulatory mechanisms underlying IAA- and BR-responsive bending of lamina joints.

## Materials and methods

### Plant materials and growth conditions

A new allele of *lpa1* (*lpa1-2*) was isolated from gene trap *Ds* populations of the Dongjin japonica rice cultivar. For the wild types (WTs), either Dongjin or WT siblings from segregation populations were used. The *d61* and *d2* mutants (japonica type) were obtained from Dr Koh Hee Jong of Seoul National University. Following a previously published protocol (Chin *et al.*, 1999), transgenic lines were generated from japonica rice cv. Dongjin. Plants were grown in paddy fields from June to October in South Korea and in the greenhouse during the winter.

### Lamina inclination assay

Plants were grown on half-strength Murashige and Skoog (MS) medium in a growth chamber at 30°C under a 24h light regime. For the lamina inclination assays, 1 µl of ethanol containing a series of concentrations of BRs, IAA or Brz was spotted onto the tips of the lamina of 5- or 6-day-old seedlings. C-22-hydroxylated and 6-deoxo brassinosteroids were identical to those described by Nakamura *et al.* (2009). Treated seedlings were grown for two additional days, and lamina joints of the second leaves were photographed for lamina bending angle measurements.

### Cloning, vector construction and transformation

Analysis of *LPA1* cDNA from wild type and trap *Ds* line, vector construction, and transformation were performed following standard methods. See Supplementary Appendix S1, available at *JXB* online, for details.

### Expression analysis

Total cellular RNA was isolated with TRIZOL reagent (MRC, http://www.mrcgene.com/) or an RNeasy Plant Mini Kit (Qiagen). For quantitative RT-PCR, the published methods of Je *et al.* (2010) were followed. The qRT-PCR products were quantified using CFX Manager software (Bio-Rad) and the values were normalized against 25S rRNA and Ubiquitin cDNA from the same samples.

### RNA-seq analysis

Using an RNeasy Plant Mini Kit (Qiagen, http://www.qiagen.com/), total RNA was extracted from 1 cm-long leaf segments spanning the second lamina joints of 1-week-old seedlings of WT, mutants and overexpressors. Construction and sequencing of cDNA libraries for RNA sequencing and data analysis were described in Supplementary Appendix S2.

### IAA treatments

Seeds were plated on half-strength MS agar medium and incubated in a growth chamber at 28°C under continuous light for 1 week. Uniform seedlings were selected and gently washed in dH_2_O to remove residual MS agar from their roots. For IAA treatments, seedling samples were transferred into 50ml Falcon tubes filled with distilled water. After acclimatization for 1 d, the samples were transferred to new Falcon tubes filled with 50ml of 20 µM IAA solution. After 3h of submergence, 1 cm-long segments that contained joints between the blades and sheaths of second leaves were sampled from 30 *lpa1*, e19 and WT plants. Using an RNeasy Plant Mini Kit (Qiagen, http://www.qiagen.com/), total RNA was extracted from leaf segments before and after 3h of IAA treatment.

### GUS staining

GUS activity was detected by incubation in a 5-bromo-4-chloro-3-indoyl glucuronide solution, which was previously described (Chin *et al.*, 1999). Samples were incubated at 37°C for 2 d in the dark and then dehydrated in a 30–70% graded ethanol series.

### Microscopy

To detect GFP fluorescence, roots of *LPA1*:*GFP* transgenic plants were analysed using an Olympus confocal laser-scanning microscope (Fluoview FV 1000, http://www.olympus-global.com/). Fluorescence emission images were collected in the 480–540nm range. Propidium iodide (PI) staining was used for detection of nuclei.

 For cellular measurement of lamina joint tissues, lamina joint tissues were collected from 1-week-old seedlings and fixed in 70% ethanol. The epidermal layers on the adaxial sides were imaged by microscopy (DP70; Olympus, Japan), and then the cell dimensions were measured by ImageJ.

## Results

### The identification of a new LPA1 allele, lpa1-2

**Fig. 1.**
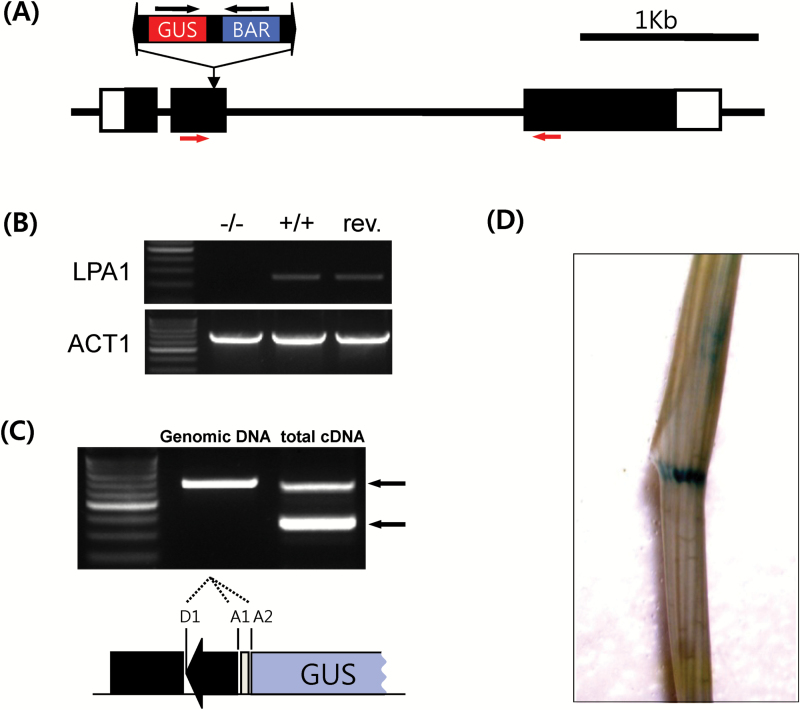
Analysis of the *lpa1::Ds* mutant allele and the expression pattern of *LPA1.* (A) The gene trap *Ds* carrying a GUS coding region and a *bar* gene was inserted at the second exon of *LPA1* (LOC_Os03g13400). Open boxes indicate the untranslated region. Closed boxes indicate coding regions. Lines between boxes indicate introns. Black horizontal arrows indicate the orientation of transcription. (B) RT-PCR analysis was performed with RNAs from 2-week-old seedlings of *lpa1*, WT and a revertant. Primers recognized the second exon before the *Ds* insertion and the third exon. The locations of the primers are shown as red horizontal arrows in panel A. The Actin gene was used as a control. (C) GUS-fused transcripts were analysed by RT-PCR using primers from *LPA1* and *GUS*. The upper arrow indicates cDNA from unspliced RNAs while the lower arrow indicates cDNA from spliced RNAs. The spliced cDNAs were sequenced to identify the joint sequences between the *LPA1* and *GUS* coding regions. D1, A1 and A2 indicate splicing donor 1 and acceptor site 1 and 2, respectively, installed inside the element (Chin *et al.*, 1999). (D) Leaf of 2-week-old *lpa1:Ds* plant incubated in GUS solution. Strong GUS staining was detected in the lamina joint. (This figure is available in colour at *JXB* online.)

In 2007, we identified a mutant line with wide lamina and tiller angles in a *Ds* population field. This mutant arose from insertion of a gene trap *Ds* at the second exon of the gene *LPA1* (Fig. 1A). RT-PCR showed that transcripts beyond the *Ds* insertion site were not detected in the mutants (Fig. 1B). Since a GUS coding region inside the gene trap *Ds* element was in the same orientation as the gene, GUS was to be transcribed with the 5ʹ truncated mRNA (Chin *et al.*, 1999). We cloned the fusion transcripts by RT-PCR, and subsequent sequencing analysis revealed that two-thirds of the spliced transcripts were in-frame between the gene and GUS (Fig. 1C). New alleles including revertants were generated by remobilizing *Ds* via tissue culture. Of the more than 100 regenerated plants, four frame-shift alleles and three revertants were identified by sequencing *Ds* excision sites (Supplementary Table S1). *LPA1* (Wu *et al.*, 2013) is the same gene that was previously reported as *OsIDD14* (Colasanti *et al.*, 2006). Therefore, this *Ds*-tagged allele was designated as *lpa1-2* and utilized for this study. LPA1/OsIDD14 has the highest sequence homology to SGR5/AtIDD15 of Arabidopsis (Morita *et al.*, 2006), and it shares 42% identity and 54% similarity with SGR5/AtIDD15 at the amino acid level. *LPA1* was highly expressed in lamina joints (Wu *et al.*, 2013). To confirm the preferential expression of *LPA1* at lamina joints, we examined GUS expression inside the gene trap *Ds* within *LPA1* (*LPA1::Ds*). The GUS pattern of *LPA1::Ds* was consistent with the expression pattern detected by qRT-PCR. Strong GUS activity was detected in the lamina joint (Fig. 1D).

### LPA1 as a positive regulator suppressing lamina bending

**Fig. 2.**
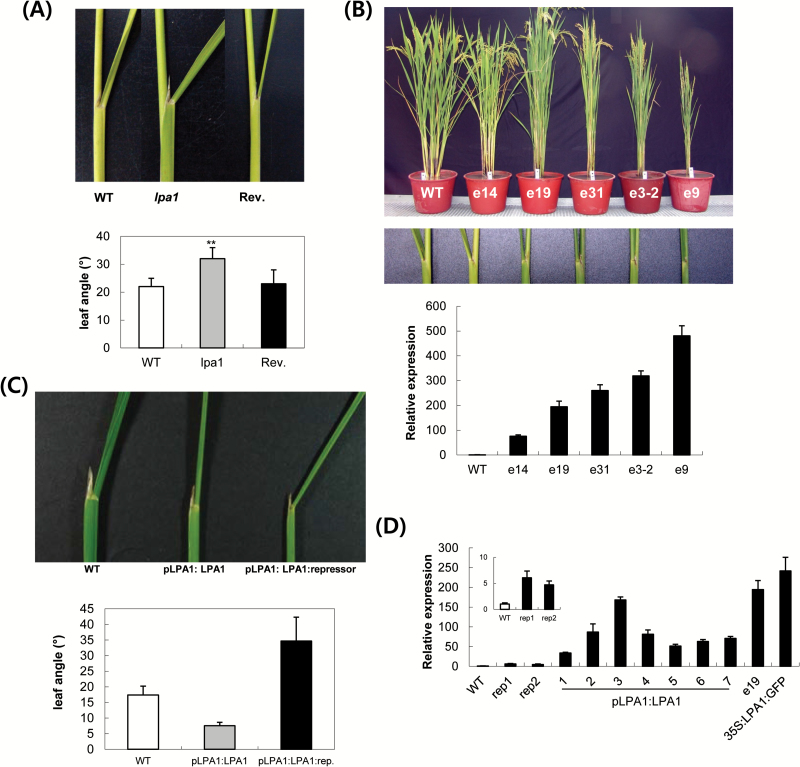
Phenotypic analysis of *lpa1*, *LPA1* overexpressors (OX) and *pLPA1:LPA1:repressor* transgenic plants. (A) At the top, lamina joints of the leaves just below flag leaves at the reproductive stage are compared among WT, *lpa1* and a revertant. At the bottom, measurements of lamina joint angles are shown in the diagrams. Data represent mean ±SE of more than ten plants analysed. **, *P*<0.001; *P*-value of *lpa1* mutants was calculated with respect to one of WT or of revertant (B) OX lines (*e14*, *e19*, *e31*, *e3-2* and *e9*) were aligned based on the expression levels of *LPA1* mRNA. Lamina joint angles are shown at the reproductive stage. Using RNA from 10-day-old seedlings, qRT-PCR was employed to measure the expression levels of individual OX lines. Values were normalized against *Ubiquitin* cDNA from the same samples. (C) The third leaves from the reproductive stage plants are compared among Dongjin parental line (WT), *pLPA1:LPA1* and *pLPA1:LPA1:repressor*. Since independent transgenic lines of the same constructs showed similar phenotypes of lamina bending, representative phenotypes are shown in the figure. At the bottom, measurements of lamina joint angles are shown in the diagrams. Data represent mean ±SD of more than ten plants from the lines analysed. (D) Levels of *LPA1* mRNA of seven *pLPA1:LPA1* (#1–7) and two *pLPA1:LPA1:repressor* (#1 and 2) lines measured by qRT-PCR using RNAs from 10-day-old seedlings. Values were normalized against *Ubiquitin* cDNA from the same samples. The transgenes were expressed under the control of the 2.4kb *LPA1* promoter. (This figure is available in colour at *JXB* online.)

We examined the mode of action of *LPA1* on lamina bending by comparing the phenotypes among knockout, overexpression and repressor-fusion transgenic plants (Fig. 2). All of the mutants, overexpression and repressor-fusion transgenic lines were generated in the same rice japonica variety, Dongjin. The *Ubiquitin* promoter was used to overexpress *LPA1* cDNA. To construct a vector expressing repressor-fused LPA1, the C-terminus of LPA1 was fused with the 24 amino acid fragment containing a conserved ERF-associated amphiphilic repression (EAR) motif of a gene (LOC_Os02g01090) orthologous to Arabidopsis *SUPERMAN* (At3g23130) (Hiratsu *et al.*, 2004). The fusion gene was expressed under the control of the 2.4kb *LPA1* promoter. We examined the expression levels of these lines by qRT-PCR.

 Lamina angles in *LPA1*-overexpressing plants and *lpa1* mutants were not significantly different from those of WT plants for up to one month of growth, even under field conditions. After one month of growth, the *Ds* mutants exhibited exaggerated leaf angles, while the overexpressor lines exhibited an erect phenotype, with narrow leaf angles (Fig. 2A, B). The extent of the leaf angle was negatively correlated with the expression level of *LPA1* mRNA in the overexpressors (Fig. 2B); plants expressing higher levels of mRNA exhibited more severe erect phenotypes. Higher *LPA1* mRNA levels were associated with slightly shorter plant statures than those of WT plants, and these plants produced fewer tillers and narrower leaves. However, flowering time was not affected. Therefore, the function of *LPA1* might be related to the suppression of lamina inclination. To further understand whether LPA1 acts as a positive or negative regulator in suppressing lamina inclination, we examined transgenic plants expressing repressor-fused *LPA1* cDNA under the control of the 2.4kb *LPA1* promoter. As a control, *LPA1* cDNA was expressed under the control of the same *LPA1* promoter. Figure 2C shows the lamina bending phenotype of the repressor-fusion LPA1 plants. The phenotype of the repressor-fused LPA1 plants exhibited the exaggerated lamina angles as observed in the knockout mutants (*lpa1*). By contrast, the *pLPA1*:*LPA1* transgenic plants accumulated higher levels of *LPA1* mRNA than normal plants (Fig. 2D) and developed the same erect phenotype as overexpressors under control of the *Ubiquitin* promoter. The data clearly show that the loss-of-function of *LPA1* led to an increase in lamina joint bending angles. Therefore, LPA1 acts as a positive regulator that suppresses lamina bending.

### The suppressive action of LPA1 on IAA-mediated lamina inclination is influenced by BR

**Fig. 3.**
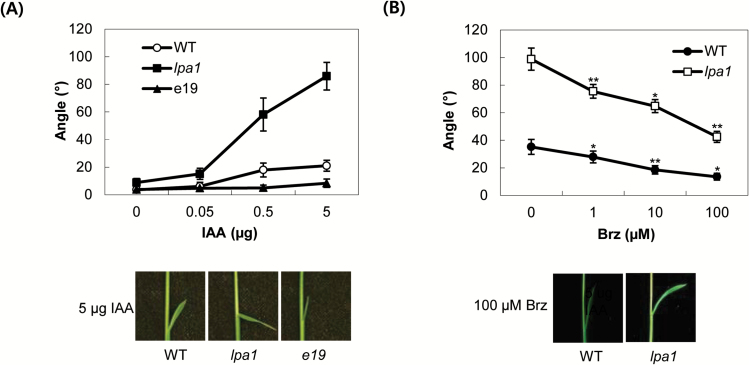
IAA-mediated lamina inclination and the effect of brassinazole on *LPA1* mutants. (A) The indicated amounts of IAA in 1 µl of ethanol were spotted onto the tips of the second lamina of 5-day-old seedlings. After 2 d of incubation, the lamina joints were photographed for angle measurements; e19 is an *LPA1* overexpressor line. A composite photograph of plants treated with aliquots of 5 µg IAA was taken. Mean values were derived from measurements of at least ten plants. Data represent the means ±SD of at least ten plants. (B) Five-day-old *lpa1* and WT plants were treated with the indicated concentrations of brassinazole (Brz) on the tips of the second leaves 12h before 5 µg IAA treatment. Data represent the means ±SD of at least ten plants. *, *P*<0.05; **, *P*<0.001; *P*-values of samples treated with 1, 10 and 100 µM Brz were calculated with respect to those of the same samples treated with 0, 1 and 10 µM Brz, respectively. (This figure is available in colour at *JXB* online.)

To examine whether *LPA1* affects IAA-mediated lamina inclination, we measured the IAA sensitivity of lamina joints in the *lpa1* mutant and an *LPA1* overexpression line, e19. During the seedling stages, the angles of lamina joints were not obviously different among WT, mutant and overexpressor. We applied a series of different concentrations of IAA to the lamina joints of 5-day-old plants grown on half-strength MS medium (Fig. 3A and Supplementary Fig. S1A) and measured the downward inclination angles 2 d after treatment. Compared with WT plants, *lpa1* plants were exceptionally sensitive to auxin. In the 5 µg IAA treatment group, WT sibling plants exhibited 20–40° angles in their lamina joints, while those of *lpa1* plants were 100°. By contrast, the overexpressor was almost completely insensitive to auxin. These results imply that *LPA1* exerts inhibitory action on auxin-mediated lamina inclination. To determine whether BRs influence the IAA-hypersensitivity of *lpa1* plants, we pretreated these plants for 12h with a series of concentrations of brassinazole (Brz) prior to the application of 5 µg IAA. Brz inhibits C-22 hydroxylase in the BR synthesis pathway and blocks the production of C-22-hydroxylated BRs (Supplementary Fig. S7; Asami *et al.*, 2001; Sakamoto *et al.*, 2006). Figure 3B and Supplementary Fig. S1B show a comparison of the Brz sensitivity of lamina joints between *lpa1* and the WT. As the concentration of Brz increased, auxin-mediated bending was suppressed in the *lpa1* lamina joints. These data clearly show that BRs are involved in the hypersensitivity of *lpa1* to IAA.

### Synergic effects of lpa1 with d2, but not OsBRI1, on auxin-mediated lamina inclination

**Fig. 4.**
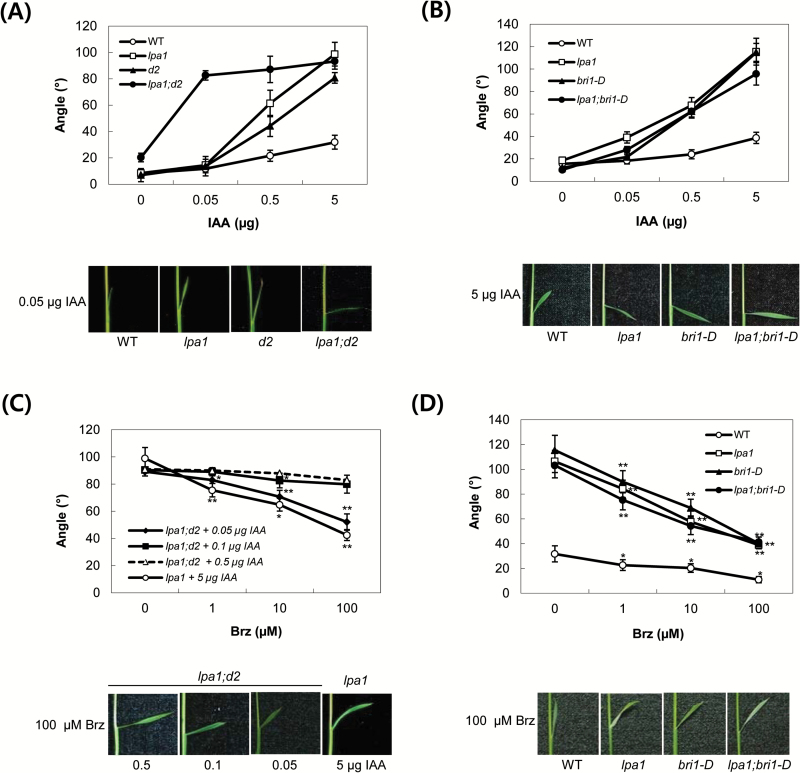
IAA-mediated lamina inclination and the effect of brassinazole on *lpa1;d2* and *lpa1;bri1-D.* (A, B) The indicated amounts of IAA in 1 µl of ethanol were spotted onto the tips of the second lamina of 5-day-old seedlings. The bending angles were measured after 2 d of incubation. A composite photograph of plants treated with 0.05 (A) and 5 µg IAA (B) was taken. Data represent the means ±SD of at least ten plants. (C) Five-day-old *lpa1;d2* plants were treated with the indicated concentrations of brassinazole (Brz) on the tips of the second leaves 12h before IAA treatment; 0.05, 0.1 and 0.5 µg IAA were applied to Brz-treated plants, which were incubated for 2 d before measurement. For comparison, the effect of Brz on *lpa1* plants treated with 5 µg IAA is shown. A composite photograph of plants treated with aliquots of 100 µM Brz was taken. (D) Five-day-old *lpa1*, *lpa1;bri1-D* and *bri1-D* plants were treated with the indicated concentrations of Brz on the tips of the second leaves 12h before 5 µg IAA treatment. A composite photograph of plants treated with aliquots of 100 µM Brz was taken. Data represent the means ±SD of at least ten plants. *, *P*<0.05; **, *P*<0.001; *P*-values of samples (C and D) treated with 1, 10 and 100 µM Brz were calculated with respect to those of the same samples treated with 0, 1 and 10 µM Brz, respectively. (This figure is available in colour at *JXB* online.)

There are two possible explanations for the effect of Brz treatment on IAA-mediated inclination of lamina joints in *lpa1*. One explanation is that Brz treatment decreases the levels of endogenous BRs in the synthesis pathway, such as C-22-hydroxylated and 6-deoxo BRs, which are involved in IAA-mediated lamina inclination (Nakamura *et al.*, 2009). The other explanation is that Brz treatment leads to a loss of active BR compounds, such as BL or castasterone (CS), which are required for the activity of OsBRI1. To distinguish between these two possibilities, *d2* and *bri1-D* were crossed with *lpa1* to generate double genetic combinations. Rice *D2* encodes a CYP90D2 enzyme, which catalyses C-23 hydroxylation of various C-22-hydroxylated BRs [e.g. C-22-hydroxycampesterol (22-OHCR) and 6-deoxocathasterone (6-deoxoCT)] (Supplementary Fig. S7; Hong *et al.*, 2003; Ohnishi *et al.*, 2006; Nakamura *et al.*, 2009). Therefore, *d2* has accumulation of C-22-hydroxylated BRs and has been shown to enhance the IAA sensitivity of lamina joints (Nakamura *et al.*, 2009). An *OsBRI1* overexpression line (*bri1-D*) isolated from an activation T-DNA tagging population exhibits increased lamina joint angles (Jeong *et al.*, 2002; Je *et al.*, 2010). First, we compared the lamina inclination of WT, *lpa1*, *lpa1;d2* and *d2* in response to IAA treatment; all lines were derived from crosses between *lpa1* and *d2*. Compared to *lpa1* and *d2, lpa1;d2* was extremely sensitive to IAA, achieving the maximal angles of lamina bending at only 0.05 µg IAA. For *lpa1* and *d2,* the same maximal angle was observed at 5 µg IAA. IAA sensitivity was approximately 100-fold higher in *lpa1;d2* than in each single mutant (Fig. 4A; Supplementary Fig. S2A). Second, to examine the action of *OsBRI1* in the lamina joints of *lpa1*, we compared the IAA sensitivity in the double genetic combination *lpa1;bri1-D* with that of *lpa1* and *bri1-D*. All lines were derived from crosses between *lpa1* and *bri1-D*. As shown in Fig. 4B and Supplementary Fig. S2B, the magnitude of IAA-mediated lamina bending angles in *lpa1;bri1-D* was similar to that of *lpa1* or *bri1-D* alone. These data indicate that the IAA-mediated lamina inclination of *lpa1* is influenced by BRs (especially C-22-hydroxylated BRs) rather than *OsBRI1*.

 In addition, we examined the effect of Brz on IAA-mediated lamina inclination in double genetic combinations *lpa1;d2* and *lpa1;bri1-D.* For *lpa1;d2*, Brz treatment had much less of an effect on IAA-mediated inclination. To achieve the same magnitude of the Brz effect as observed in *lpa1* and *d2*, *lpa1;d2* plants required 100-fold lower IAA concentrations (Fig. 4C; Supplementary Fig. S2C). Such a reduction in the effect of Brz on *lpa1;d2* may have been due to an interaction between *lpa1* and *d2* in the lamina joints that occurred prior to Brz treatment. By contrast, Brz had a similar effect on IAA-mediated inclination in *lpa1;bri1-D* to that in *lpa1*. Under increasing levels of Brz treatment, *lpa1* and *lpa1;bri1-D* showed similar rates of inhibition of auxin-mediated bending of lamina joints (Fig. 4D; Supplementary Fig. S2D).

### Sensitivity to C-22-hydroxylated and 6-deoxo BRs during lamina inclination

**Fig. 5.**
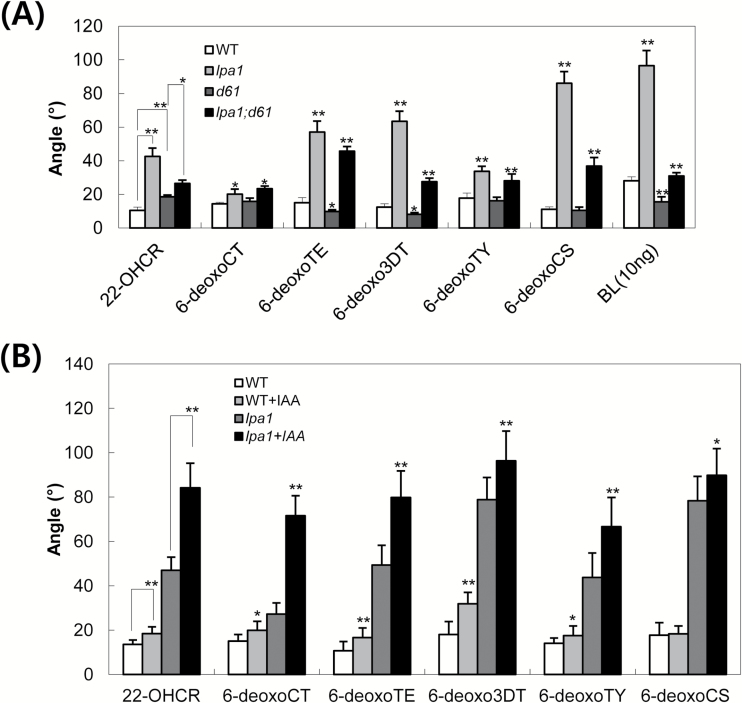
Sensitivity of *lpa1* and *lpa1;d61-1* plants to C-22-hydroxylated and 6-deoxo brassinosteroids (BRs) and the combinatory effects with IAA on *lpa1* mutants. (A) Aliquots of 100ng each of 22-OHCR, 6-deoxoCT, 6-deoxoTE, 6-deoxo3DT, 6-deoxoTY, 6-deoxoCS and 10ng BLs were applied to 5-day-old plants for 2 d and the resultant bending angle of the lamina joint was measured. Data represent the means ±SD of at least ten plants. *, *P*<0.05; **, *P*<0.001; *P*-values of *lpa1*and *d61-1* were calculated with respect to the WT samples. The *P*-value of *lpa1;d61-1* was calculated with respect to *d61-1*. (B) Mixtures of 0.05 µg IAA and 100ng aliquots each of 22-OHCR, 6-deoxoCT, 6-deoxoTE, 6-deoxo3DT, 6-deoxoTY and 6-deoxoCS were applied to 5-day-old plants for 2 d and the bending angles of lamina joints were measured. Data represent the means ±SD of at least ten plants. *, *P*<0.05; **, *P*<0.001; *P*-values of samples treated only with BR were calculated with respect to those treated with both IAA and the same BR. 22-OHCR, C-22-hydroxycampesterol; 6-deoxoCT, 6-deoxocathasterone; 6-deoxoTE, 6-deoxoteasterone; 6-deoxo3DT, 3-dehydro-6-deoxoteasterone; 6-deoxoTY, 6-deoxotyphasterol; 6-deoxoCS, 6-deoxocastasterone.

To verify that the IAA-mediated lamina inclination of *lpa1* was enhanced in the presence of C-22-hydroxylated BRs, we examined the sensitivity of lamina joints to C-22-hydroxylated and 6-deoxo BRs in *lpa1* and *lpa1;d61-1* (Fig. 5A; Supplementary Fig. S3A). The following compounds were directly applied to lamina joints: 22-OHCR, 6-deoxoCT, 6-deoxoTE (teasterone), 6-deoxo3DT (3-dehydroteasterone), 6-deoxoTY (typhasterol) and 6-deoxoCS. Among these BRs, 22-OHCR and 6-deoxoCT are substrates for *D2*-encoded 23-hydroxylase (Supplementary Fig. S7). The compounds 6-deoxoTE, 6-deoxo3DT, 6-deoxoTY and 6-deoxoCS are converted to TE, 3DT, TY and CS, respectively, by *CYP85A1* (*BRD1* (*BR-deficient Dwarf1*)/*OsDWARF1*)-encoded C-6 oxidase (Supplementary Fig. S7; Hong *et al.*, 2002). Due to the possibility that these C-22-hydroxylated or 6-deoxo BRs might be converted into the active BRs, CS or BL, it was important to compare the lamina sensitivity of *lpa1* with *lpa1;d61-1*. All of the lines were derived from crosses between *lpa1* and *d61-1*. As previously reported (Nakamura *et al.*, 2009), lamina bending of WT and *d61-1* plants was barely observed at a 100ng concentration of any of the BRs. However, *lpa1* exhibited clear lamina bending under these conditions. Moreover, *lpa1;d61-1* exhibited much stronger responses to all of the compounds than *d61-1*. Since lamina bending was more exaggerated in *lpa1* than in *lpa1;d61-1*, it is possible that a portion of the exogenously supplied BRs was converted to active BRs, which would bind to OsBRI1 in the cells of lamina joints, thereby explaining the difference in lamina inclination between *lpa1* and *lpa1;d61-1*. Taken together, these results suggest that *LPA1* enhances the sensitivity of lamina joints not only to C-22-hydroxylated BRs, but also to deoxo BRs, which is independent of the action of *OsBRI1*.

 To estimate the effect of C-22-hydroxylated and 6-deoxo BRs on IAA-mediated lamina bending, we applied 100ng each of 22-OHCR, 6-deoxoCT, 6-deoxoTE, 6-deoxo3DT, 6-deoxoTY and 6-deoxoCS, along with 0.05 µg IAA, to lamina joints of *lpa1* and WT plants. The IAA dosage of 0.05 µg was chosen since lamina bending was not evident in mutant or WT plants treated with 0.05 µg IAA (Fig. 3A). Figure 5B and Supplementary Fig. S3B show that significant interactions of these BRs with IAA were detected in *lpa1*. It is worth noting that the plants were more sensitive to 6-deoxo3DT, 6-deoxoTY and 6-deoxoCS than to 22-OHCR or 6-deoxoCT, which may be due to the fact that 6-deoxo3DT, 6-deoxoTY and 6-deoxoCS are substrates that are directly converted to CS or BL, which bind to OsBRI1. Since the maximal bending angle of lamina joints is approximately 100°, the combined effects of these 6-deoxo BRs and IAA on lamina bending were not as dramatic as expected. Taken together, these results suggest that *LPA1* exerts a regulatory role in determining lamina bending via the interaction of IAA with C-22-hydroxylated and 6-deoxo BRs.

### Relationship between LPA1 and OsBRI1 in determining lamina inclination

**Fig. 6.**
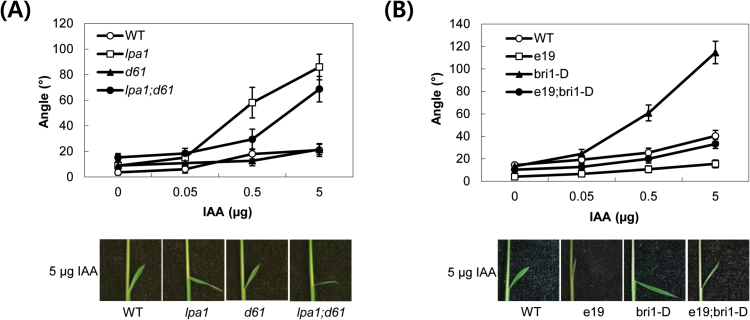
IAA-mediated lamina inclination of *lpa1;d61-1* and *e19;bri1-D*. The indicated amounts of IAA in 1 µl of ethanol were spotted onto the tips of the second lamina of 5-day-old seedlings. All lines were derived from crosses of *lpa1* with (A) *d61-1* and (B) *bri1-D*. Data represent the means ±SD of at least ten plants. (This figure is available in colour at *JXB* online.)

The data strongly suggest that *LPA1* and *OsBRI1* exert independent effects on IAA-mediated lamina inclination. As shown in Fig. 4B, both *lpa1* and *bri1-D* were hypersensitive to IAA and showed similar bending angles in response to IAA in the lamina inclination assay. To further examine the relationship between *LPA1* and *OsBRI1* in auxin-mediated lamina inclination, two double genetic combinations of *lpa1;d61-1* and *e19;bri1-D* were utilized. As shown in Fig. 6A and Supplementary Fig. S4A, the extent of lamina inclinations in *lpa1;d61-1* was intermediate between that of *lpa1* and *d61-1*. Similarly, the bending angles of *e19;bri1-D* were similar to those of the WT and were intermediate between those of *e19* and *bri1-D* (Fig. 6B and Supplementary Fig. S4B). Therefore, *LPA1* and *OsBRI1* are independently required for IAA-mediated lamina inclination (Supplementary Fig. S5).

### Cellular measurements of the epidermal layer of lamina joints

**Fig. 7.**
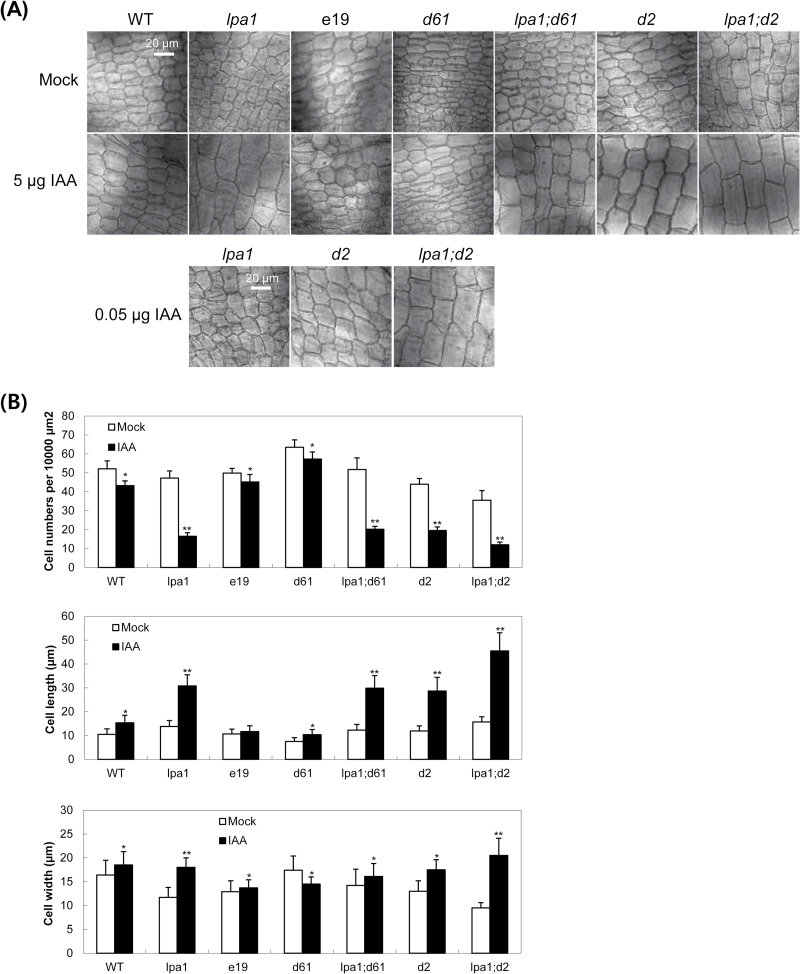
Cellular measurements in the epidermal layers of lamina joints. (A) Epidermal cells of adaxial layers of the lamina joints are shown. The lamina joints of WT (siblings from *lpa1;d61-1* segregation), *lpa1*, *e19*, *d61-1*, *lpa1;d61-1*, *d2* and *lpa1;d2* plants were treated with 1 µl ethanol (mock) or 1 µl ethanol containing 5 µg IAA. In addition, *lpa1*, *d2* and *lpa1;d2* plants treated with 0.05 µg IAA were compared. Bar, 20 µm. (B) The cell numbers, and length and width of epidermal cells of the lamina joints in these plants were measured by ImageJ after being imaged by microscopy. Data represent the means ±SD of at least four independent plants. *, *P*<0.05, **, *P*<0.001; student’s *t*-test. *P*-values of samples treated with IAA were calculated with respect to those of non-treated samples.

To obtain detailed information about the cellular responses of *LPA1*-affected lamina joints, we measured the cellular dimensions in the epidermal layers on the adaxial sides of these joints (Fig. 7A). We compared the lamina joints before and 2 d after 5 µg IAA treatment among *lpa1*, e19 (an *LPA1* OX line), *d61-1*, *lpa1;d61-1*, *lpa1;d2*, *d2* and WT siblings from the cross between *lpa1* and *d61-1*. We also compared *lpa1;d2* with *lpa1* and *d2* plants treated with 0.05 µg IAA. Figure 7B shows the cellular parameters of epidermal layers of the adaxial side. Cell numbers within given areas were dramatically reduced in *lpa1*, *lpa1;d61-1*, *lpa1;d2* and *d2* after the IAA treatments. Likewise, they showed significant increases in cell length in the same areas. Interestingly, *lpa1* and *lpa1;d2* exhibited enhanced expansion of cell width compared to *lpa1;d61-1* and *d2*. Therefore, the dramatic lamina inclination observed in *lpa1;d2* may have resulted from increases in both cell width and length.

### Possible mechanisms underlying the role of LPA1 in IAA-mediated lamina inclination

**Fig. 8.**
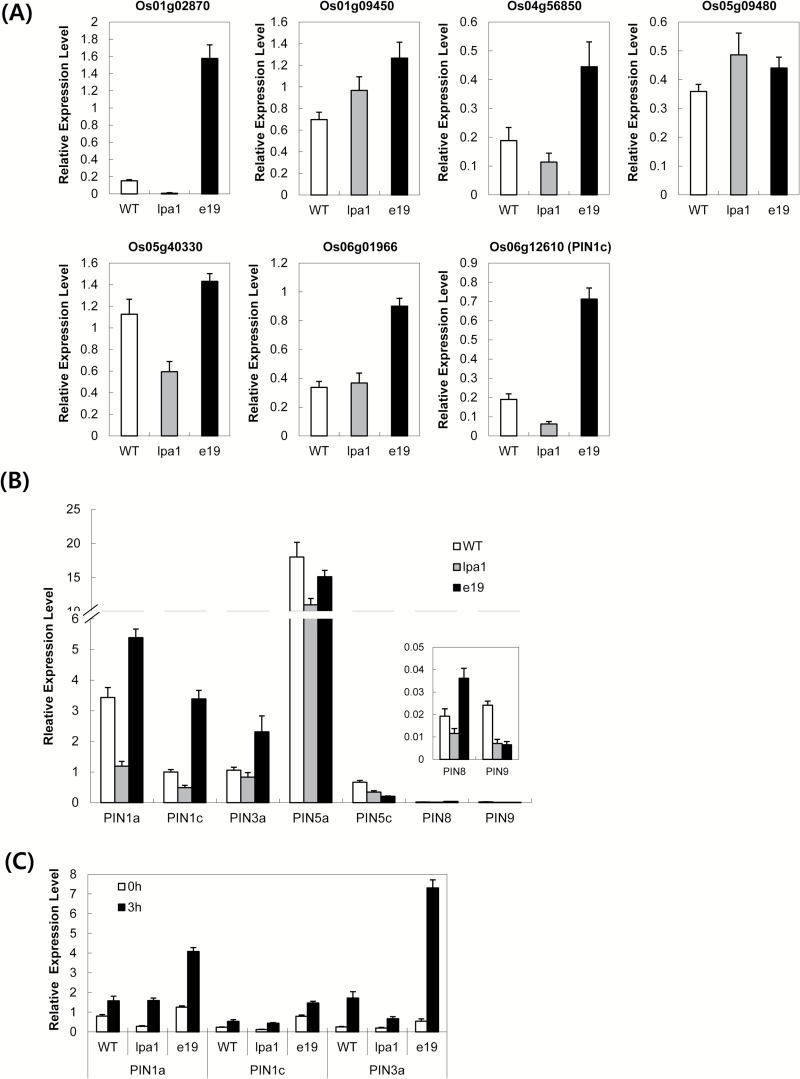
Quantitative RT-PCR analysis of auxin-related genes identified by RNA-seq analysis and *OsPIN* genes in WT, *lpa1* and e19. RNA from 1 cm-long leaf segments spanning the lamina joints of WT, *lpa1* and e19 was used for qRT-PCR analysis. (A) The expression of LOC_Os01g02870, LOC_Os06g12610, LOC_Os04g56850, LOC_Os05g09480, LOC_Os01g09450, LOC_Os06g01966, and LOC_Os05g40330, whose putative functions are listed in Supplementary Table S4, was re-examined by qRT-PCR. (B) The expression levels of seven *OsPIN* genes (*OsPIN1a*, *OsPIN1c*, *OsPIN3a*, *OsPIN5a*, *OsPIN5c*, *OsPIN8* and *OsPIN9*) were measured by qRT-PCR. (C) Quantitative RT-PCR was performed to estimate the mRNA levels of *OsPIN1a*, *OsPIN1c* and *OsPIN3a* in WT, *lpa1* and e19 before and 3h after IAA treatment; 25S rRNA was used as a control to normalize the expression data. Error bars are ±SD of the means of three qPCR replicates.

To explore the molecular mechanism underlying how *LPA1* is involved in IAA-mediated lamina inclination, transcriptome profiling using Illumina RNA-seq was performed on leaf segments spanning lamina joints from *lpa1* mutant, overexpressor (e19) and WT siblings. Total RNA was isolated from 1 cm-long leaf segments that spanned the lamina joints of 10-day-old plants. After analysis of RNA-seq reads, genes were identified that showed at least 1.5-fold differences in expression levels between WT sibling and *lpa1* mutant or e19 overexpressor plants. Supplementary Table S3 lists the genes whose expression was 1.5-fold higher or lower in e19 or *lpa1* than in the WT. Among these genes, seven genes whose functions are related to auxin signalling or action were further analysed using quantitative RT-PCR (Supplementary Table S4 and Fig. 8A). Among the auxin-related genes, four genes showed opposite expression patterns between *lpa1* and e19. Compared to WT, these genes were downregulated in *lpa1* and upregulated in e19. Particularly LOC_Os06g12610, which belongs to the *OsPIN* gene family and encodes the auxin efflux carrier OsPIN1c. To further evaluate the effect of *LPA1* on the expression of *OsPIN* family genes, we examined RNA from leaf segments spanning lamina joints of *lpa1*, e19 and WT siblings using qRT-PCR (Fig. 8B). Twelve *OsPIN* genes are reported to be expressed in rice (Miyashita *et al.*, 2010). The transcripts of seven genes were analysed by qRT-PCR using previously reported primers (Miyashita *et al.*, 2010). Four *OsPIN* genes (*OsPIN1a*, *OsPIN1c*, *OsPIN3a* and *OsPIN5*) produced major RNA species in these leaf samples. Among these, three *OsPIN* genes (*OsPIN1a*, *OsPIN1c* and *OsPIN3a*) exhibited *LPA1*-dependent expression patterns; these genes were expressed at lower levels in *lpa1* and higher levels in e19. To examine whether *LPA1* influences the auxin sensitivity of these three *OsPIN* genes, we compared the RNA levels of *OsPIN1a*, *OsPIN1c* and *OsPIN3a* before and 3h after IAA treatment. Even after IAA treatment, the mutants maintained lower transcript levels of these three genes than the WT siblings, while the overexpressors produced higher transcript levels (Fig. 8C). These results indicate that the auxin-inducibility of these *OsPIN* genes is influenced by *LPA1*.

## Discussion

The functions of *LPA1* and its Arabidopsis homologues have been elucidated in previous reports. *LPA1* and *SGR5/AtIDD15* have defects in shoot gravity responses; therefore, they have been proposed to function during early shoot gravitropism responses by regulating amyloplast sedimentation rates (Morita *et al.*, 2006; Wu *et al.*, 2013). Subsequent work showed that *AtIDD14*, *AtIDD15* and *AtIDD16* gene products directly target auxin biosynthetic and transport genes, and influence organ development, which eventually controls plant architecture (Cui *et al.*, 2013).

Lamina inclination is determined by cellular expansion of the lamina joint adaxial layers (Maeda, 1965). Lamina joints respond rapidly to extracellular hormones, which renders this system a suitable model for efficient analysis of cellular mechanisms regulating lamina inclination. The major objective of this study was to identify the role of *LPA1* in hormone-dependent determination of lamina inclination in rice plants. We utilized BR synthetic and receptor mutants (*d2*, *d61-1* and *bri1-D*) and treatment with IAA and BR compounds to elucidate the role of *LPA1* in interacting with auxin and BR signalling, which determined lamina bending. Our studies revealed three primary observations. First, lamina joints in *lpa1* mutants responded dramatically to IAA, which was suppressed by Brz inhibition of C-22 hydroxylase in the BR biosynthetic pathway. Second, IAA sensitivity of *lpa1* mutants was strongly enhanced in genetic combination with *d2* mutants, which are defective in CYP90D2 catalysis of C-22-hydroxylated BRs. However, no additive effect was observed in *lpa1* plants in genetic combination with the *BRI1* dominant mutation *bri1-D*. Third, *lpa1* mutants exhibited sensitivity to C-22-hydroxylated and 6-deoxo BRs even in the absence of *OsBRI1*. These combined results indicate that *LPA1* suppresses auxin signalling interacting with C-22-hydroxylated or 6-deoxo BRs, which regulates lamina inclination independently of *OsBRI1*. Supplementary Tables S5, S6 summarize the genetic and biochemical data on the auxin sensitivity of lamina bending.

Our work provides genetic evidence verifying the previously proposed hypothesis that BR interacts with auxin to determine lamina inclination via two different pathways. In one pathway, BRs interact directly with auxin. In the other pathway, the OsBRI1 receptor is required for BR interaction with auxin. Our study utilized two genetic combinations (*e19* and *bri1-D*; *lpa1* and *d61-1*) to investigate rice lamina inclination, and the results indicate that two pathways might exert independent effects on IAA-mediated lamina inclination. Currently, little is known about BR and IAA interactions in determining lamina inclination in rice, except for anatomical evidence that BR and IAA interaction results in cellular elongation and expansion (Fig. 7; Maeda, 1965; Nakamura *et al.*, 2009). Further analysis of *LPA1* would increase our understanding of lamina joint responses to auxin and BR.

Although the mechanisms underlying asymmetric responses of adaxial and abaxial cell layers to auxin during lamina bending are currently unknown, it is reasonable to speculate that differential sensitivity to auxin and asymmetrical auxin distribution are important factors in determining lamina inclination. Expression of Arabidopsis *AtPIN1* was altered in gain-of-function and loss-of-function mutants of *AtIDD14*, *AtIDD15* and *AtIDD16*, and *IDD* genes directly activated *AtPIN1* expression (Cui *et al.*, 2013). Our RNA-seq data showed that *OsPIN* expression depends on *LPA1*. This suggests that *LPA1*-dependent *OsPIN* expression might be responsible, at least in part, for the opposite auxin sensitivity during the lamina bending response between *lpa1* and *e19* plants. *OsPIN2* overexpression in rice plants increases auxin transport from shoots to roots without altering auxin flux patterns (Chen *et al.*, 2012). Therefore, it is reasonable to speculate that *LPA1* overexpressors might have a greater capacity for transporting exogenous auxin than WT plants, and *lpa1* mutants might exhibit less efficient auxin flux. To confirm the significance of auxin flux in *LPA1*-mediated lamina bending, *lpa1* mutant and overexpressor *e19* were treated with different concentrations of N-1-naphthylphthalamic acid (NPA) at lamina joints 12h before IAA application (Supplementary Fig. S6). NPA treatment increased IAA-induced lamina bending angles in *e19*-overexpressing plants but not in *lpa1* mutants. These data support the proposal that auxin transport might be more active in *LPA1*-overexpressing plants than in *lpa1* mutants. These combined results suggest that auxin flux and distribution might be important factors underlying the role of *LPA1*-mediated auxin sensitivity in lamina inclination.

Plant architecture is one of the major phenotypic parameters that crop breeders consider when developing high yielding varieties, and lamina angle is one of the major characters that determine plant type. Since overexpression of *LPA1* leads to an erect phenotype, producing plants with the optimal expression pattern of *LPA1* would lead to the production of ideal plant types. Therefore, *LPA1* should serve as an important genetic tool that can contribute to breeding elite crop varieties. Indeed, we previously demonstrated the agricultural utility of *LPA1/OsMPT1* (Han and Choe, 2010).

## Supplementary data

Supplementary data is available at *JXB* online.

Appendix S1. Cloning, vector construction and transformation.

Appendix S2. RNA-seq analysis.

Figure S1. IAA-mediated lamina inclination and the effect of brassinazole on *lpa1* mutants.

Figure S2. IAA-mediated lamina inclination and the effect of brassinazole on *lpa1;d2* and *lpa1;bri1-D.*

Figure S3. Sensitivity of *lpa1* and *lpa1;d61-1* plants to C-22-hydroxylated and 6-deoxo brassinosteroids (BRs) and the combinatory effects with IAA on *lpa1* mutants.

Figure S4. IAA-mediated lamina inclination of *lpa1;d61-1* and *e19;bri1-D*.

Figure S5. Relationship between *LPA1* and *OsBRI1* in determining lamina inclination.

Figure S6. Effect of NPA on IAA-mediated lamina inclination in *lpa1,* overexpressor (e19) and WT.

Figure S7. Brassinosteroids (BRs) and brassinazole (Brz) utilized in this study and steps catalysed by *D2* are shown in a BR biosynthetic pathway (adapted from Nakamura *et al.*, 2009).

Table S1. Footprints after *Ds* excisions.

Table S2. Total number of reads mapped per sample.

Table S3. List of genes whose expression was 1.5-fold higher or lower in e19 or *lpa1* than in the WT.

Table S4. List of auxin-related genes identified by RNA-seq.

Table S5. Auxin sensitivity of mutants for lamina inclination.

Table S6. Relative sensitivity of *lpa1* and *lpa1;d61-1* to BR compounds for lamina inclination.

Supplementary Data
