# Supplementary material for: Loose Plant Architecture1 (LPA1) determines lamina joint bending by suppressing auxin signalling that interacts with C-22-hydroxylated and 6-deoxo brassinosteroids in rice
Source: J Exp Bot. 2016 Jan 29;67(6):1883–95. doi: 10.1093/jxb/erw002 (PMC4783368; doi:10.1093/jxb/erw002)
Supplement: Supplementary Data [file supp_67_6_1883__index.html]

 Loose Plant Architecture1 (LPA1) determines lamina joint bending by suppressing auxin signalling that interacts with C-22-hydroxylated and 6-deoxo brassinosteroids in rice — Loose Plant Architecture1 (LPA1) determines lamina joint bending by suppressing auxin signalling that interacts with C-22-hydroxylated and 6-deoxo brassinosteroids in rice — Supplementary Data 

# *Loose Plant Architecture1* (*LPA1*) determines lamina joint bending by suppressing auxin signalling that interacts with C-22-hydroxylated and 6-deoxo brassinosteroids in rice

## Supplementary Data

Data files

- Supplementary\_figures\_S1\_S7\_table\_S1\_S2\_and\_S4\_S6\_appendix\_1\_and\_2.pdf - Supplementary Data
- Supplementary\_table\_S3.xlsx - Supplementary Data
