# Supplementary material for: Loose Plant Architecture1 (LPA1) determines lamina joint bending by suppressing auxin signalling that interacts with C-22-hydroxylated and 6-deoxo brassinosteroids in rice
Source: J Exp Bot. 2016 Jan 29;67(6):1883–95. doi: 10.1093/jxb/erw002 (PMC4783368; doi:10.1093/jxb/erw002)
Supplement: Supplementary Data [file supp_erw002_Supplementary_figures_S1_S7_table_S1_S2_and_S4_S6_appendix_1_and_2.pdf]

Supplementary Data

Supplementary Figures

Figure S1. IAA-mediated lamina inclination and the effect of brassinazole on *lpa1* mutants.

Figure S2. IAA-mediated lamina inclination and the effect of brassinazole on *lpa1;d2* and *lpa1;bri1-D.*

Figure S3. Sensitivity of *lpa1* and *lpa1;d61-1* plants to C-22-hydroxylated and 6-deoxo brassinosteroids (BRs) and the combinatory effects with IAA on *lpa1* mutants.

Figure S4. IAA-mediated lamina inclination of *lpa1;d61-1* and *e19;bri1-D.*

Figure S5. Relationship between *LPA1* and *OsBRI1* in determining lamina inclination.

Figure S6. Effect of NPA on IAA-mediated lamina inclination in *lpa1,* overexpressor (e19) and wild type.

Figure S7. Brassinosteroids (BRs) and brassinazole (Brz) utilised in this study and steps catalyzed by *D2* are shown in a BR biosynthetic pathway [adapted from Nakamura *et al.* (2009)].

Supplementary Tables

Table S1. Footprints after *Ds* excisions.

Table S2. Total number of reads mapped per sample.

Table S3. List of genes whose expression was 1.5-fold higher or lower in e19 or *lpa1* than in the WT.

Table S4. List of auxin-related genes identified by RNA-Seq.

Table S5. Auxin sensitivity of mutants for lamina inclination

Table S6. Relative sensitivity of *lpa1* and *lpa1,d61* to BR compounds for lamina inclination

Appendix S1. Cloning, vector construction and transformation.

Appendix S2. RNA-Seq analysis.

Supplementary Figures and Legends

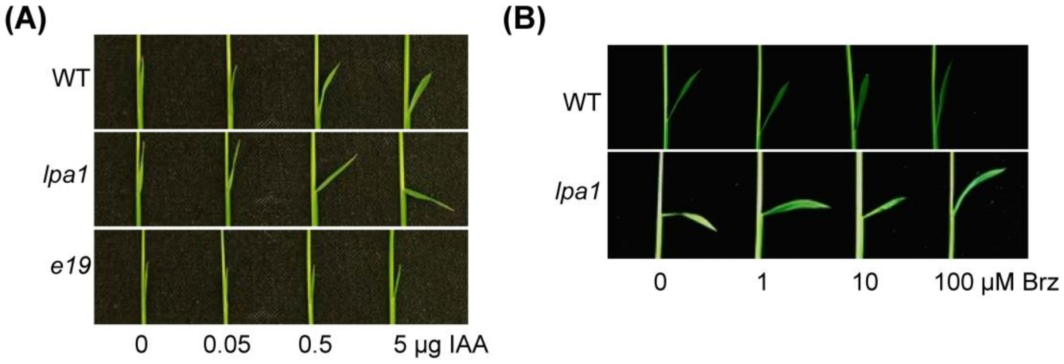

Figure S1. IAA-mediated lamina inclination and the effect of brassinazole on *lpa1* mutants.

(A) The indicated amounts of IAA in 1 μl ethanol were spotted onto the tips of the second lamina of 5-day-old seedlings. After 2 days of incubation, lamina joints were photographed for angle measurements. Line e19 is an *LPA1* overexpressor line. Typical plants from each treatment group are shown.

(B) Five-day-old *lpa1* and wild-type plants were treated with the indicated concentrations of Brz on the tips of the second leaves 12 h before IAA treatment. Typical plants from each treatments are shown.

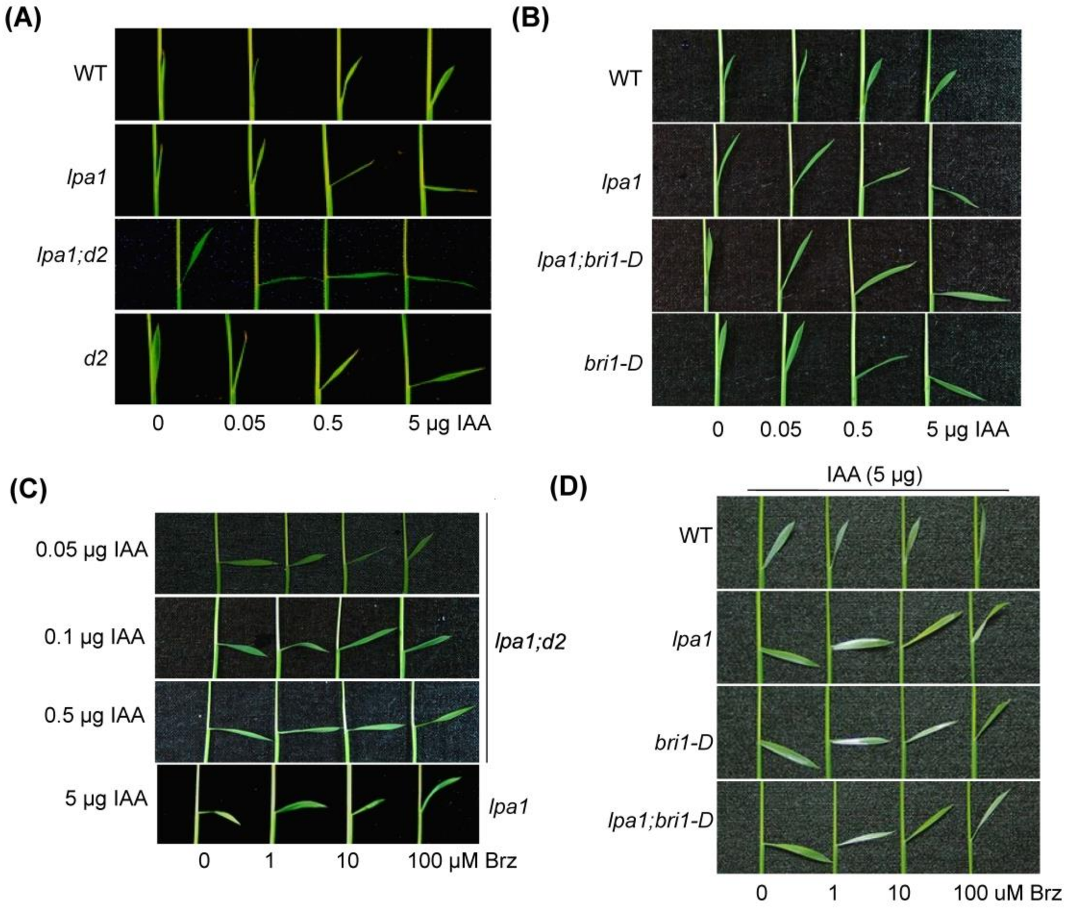

Figure S2. IAA-mediated lamina inclination and the effect of brassinazole on *lpa1;d2* and *lpa1;bri1-D.*

(A–B) The indicated amounts of IAA in 1 μl ethanol were spotted onto the tips of the second lamina of 5-day-old seedlings. The bending angles were measured after 2 days of incubation. A composite photograph was taken of plants treated with 0.05 (a) and 5 μg IAA (b). Typical plants from each treatment are shown.

(C) Five-day-old *lpa1;d2* plants were treated with the indicated concentrations of brassinazole (Brz) on the tips of the second leaves 12 h before IAA treatment; 0.05, 0.1 and 0.5 μg IAA were applied to Brz-treated plants, which were incubated 2 days before measurement. Typical plants from each treatment are shown.

(D) Five-day-old *lpa1*, *lpa1;bri1-D*, and *bri1-D* plants were treated with the indicated concentrations of Brz on the tips of the second leaves 12 h before 5 μg IAA treatment. Typical plants from each treatment are shown.

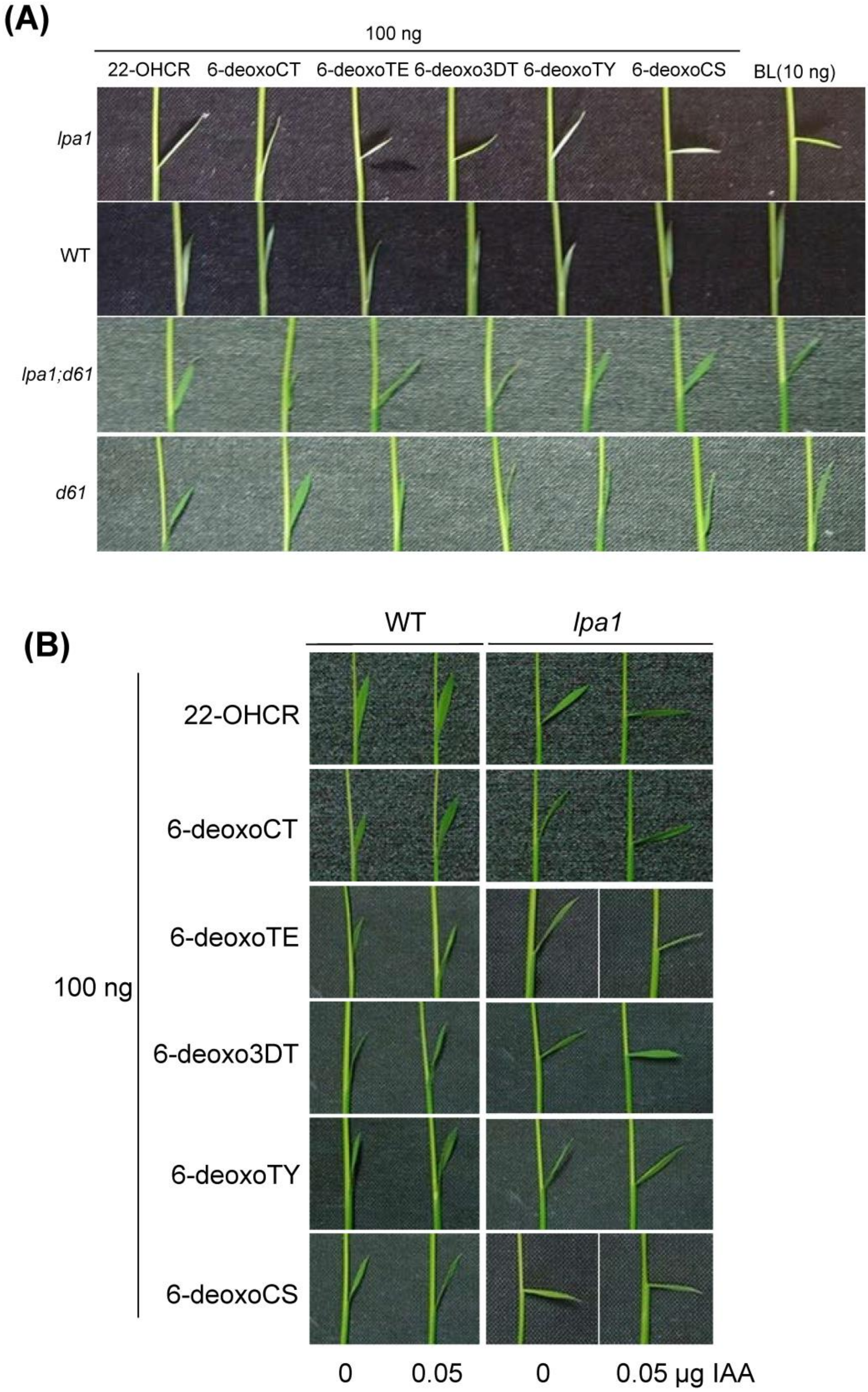

Figure S3. Sensitivity of *lpa1* and *lpa1;d61-1* plants to C-22-hydroxylated and 6-deoxo brassinosteroids (BRs) and the combinatory effects with IAA on *lpa1* mutants.

(A) Five-day-old plants were treated with 100 ng aliquots each of 22-OHCR, 6-deoxoCT, 6-deoxoTE, 6-deoxo3DT, 6-deoxoTY, 6-deoxoCS and 10 ng BL for 2 days and the resultant bending angle of the lamina joint was measured. Typical plants from each treatment are shown.

(B) A mixture of 0.05 μg IAA and 100 ng aliquots each of 22-OHCR, 6-deoxoCT, 6-deoxoTE, 6-deoxo3DT, 6-deoxoTY and 6-deoxoCS were applied to 5-day-old plants for 2 days and the bending angles of lamina joints were measured. Typical plants from each treatment are shown.

22-OHCR, C-22-hydroxycampesterol; 6-deoxoCT, 6-deoxocathasterone; 6-deoxoTE, 6-deoxoteasterone; 6-deoxo3DT, 3-dehydro-6-deoxoteasterone; 6-deoxoTY, 6-deoxotyphasterol; 6-deoxoCS, 6-deoxocastasterone.

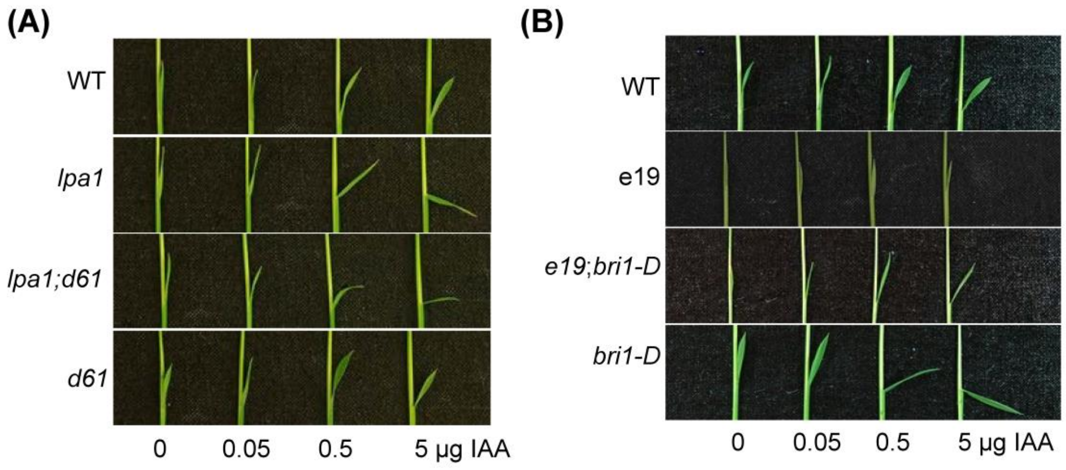

Figure S4. IAA-mediated lamina inclination of *lpa1;d61-1* and *e19;bri1-D*.

The indicated amounts of IAA in 1 μl ethanol were spotted onto the tips of the second lamina of 5-day-old seedlings. All lines were derived from crosses of *lpa1* with *d61-1* (A) and *bri1-D* (B). Typical plants from each treatment are shown.

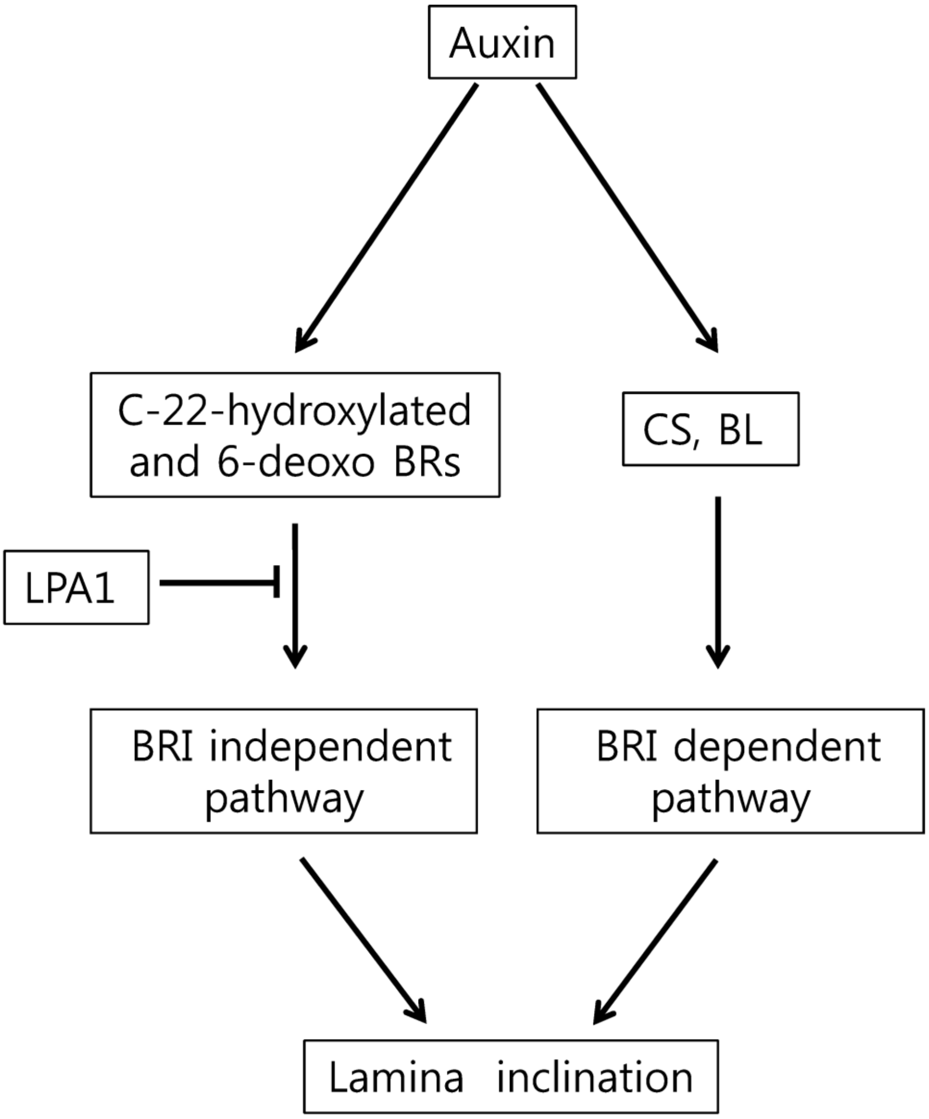

Figure S5. Relationship between *LPA1* and *OsBRI1* in determining lamina inclination

LPA1 plays a significant role in the interactive pathway of IAA and C-22-hydroxylated, 6-deoxo brassinosteroids (BRs) and exerts little influence on that of IAA and *OsBRI1* in determining lamina inclination. *LPA1* acts as a negative regulator against the C-22-hydroxylated and 6-deoxo BRs pathway.

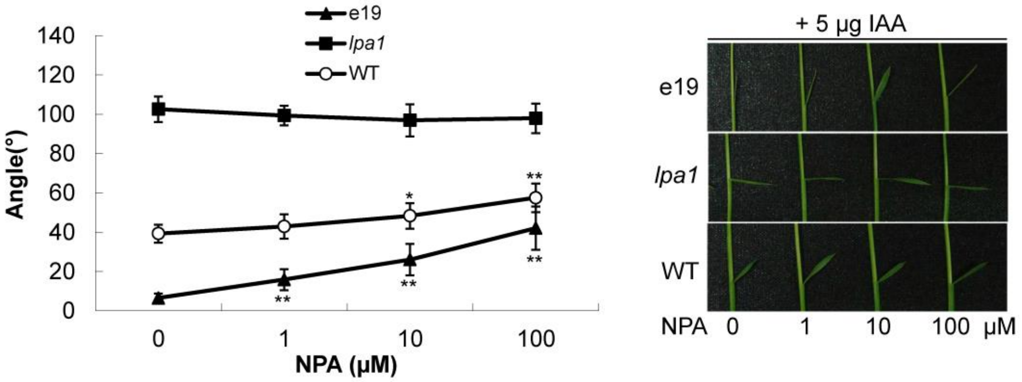

Figure S6. Effect of NPA on IAA-mediated lamina inclination in *lpa1*, overexpressor (e19) and wild type.

Five-day-old plants were treated with the indicated concentrations of *N*-1-Naphthylphthalamic Acid (NPA) on the tips of the second leaves 12 h before IAA treatment. After 12 hours, 1 µl of ethanol containing 5 µg IAA and each of the same concentrations of NPA was applied to NPA-pretreated plants, which were incubated 2 days before measurement. A composite photograph of plants treated with aliquots of the indicated concentrations of NPA was taken. Data represent the means ± SD of at least ten plants. $*P < 0.05$, $**P < 0.001$; *P*-values of samples treated with 1, 10 and 100 µM NPA were calculated with respect to those of the same samples treated without NPA (0 µM).

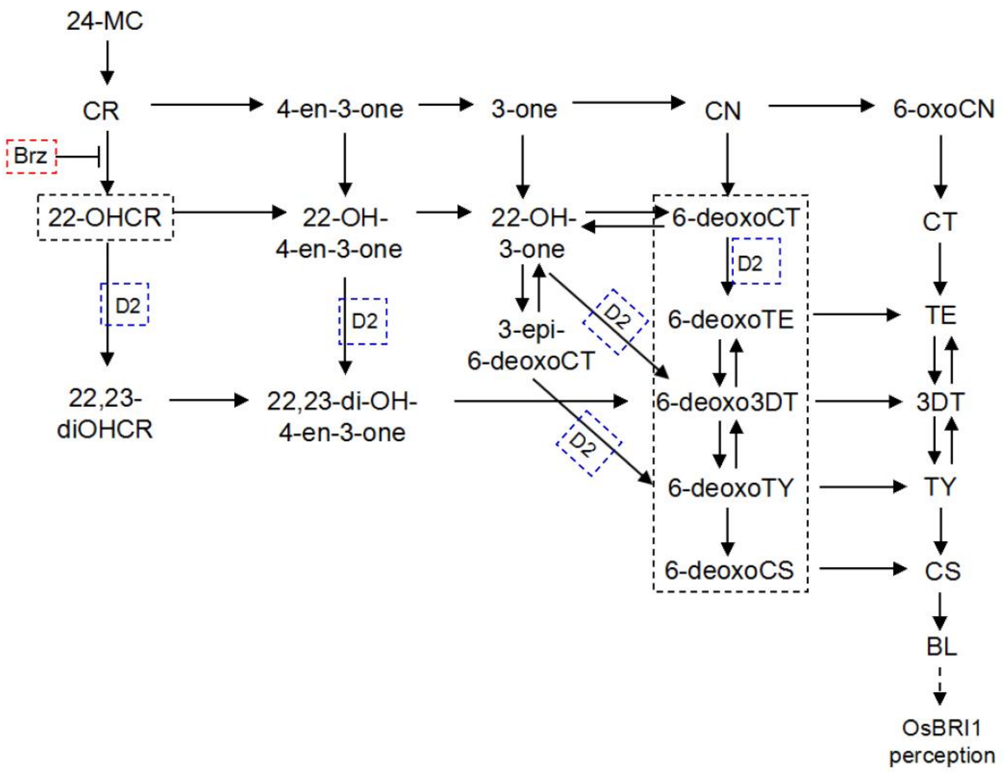

Figure S7. Brassinosteroids (BRs) and brassinazole (Brz) utilised in this study and steps catalyzed by *D2* are shown in a BR biosynthetic pathway [adapted from Nakamura *et al.* (2009)].

BRs used in this study are indicated in boxes (black dotted lines). The catalytic step inhibited by Brz was shown in with red dotted lines. *D2* Catalytic steps (labeled D2) are shown with blue dotted lines. The followings BRs were tested in this study; 22-OHCR, C-22-hydroxycampesterol; 6-deoxoCT, 6-deoxocathasterone; 6-deoxoTE, 6-deoxoteasterone; 6-deoxo3DT, 3-dehydro-6-deoxoteasterone; 6-deoxoTY, 6-deoxotyphasterol; 6-deoxoCS, 6-deoxocastasterone.

Supplementary Tables

Table S1. Footprints after *Ds* excisions.

Genotype	Revertants	Frame-shifts
Footprint sequence	gt aag a<br>gt c<br>-	gt caa ga<br>tc aag<br>tc<br>tag

Among *Ds* excision lines, footprints left after *Ds* excision were cloned and sequenced. Revertants that included a perfect excision and frame-shift mutants that included nonsense mutants are listed along with footprint sequences

Table S2. Total number of reads mapped per sample.

Genotype	Wild-type siblings		e19		*lpa1*	
Replicate	Replicate 1	Replicate 2	Replicate 1	Replicate 2	Replicate 1	Replicate 2
Total number of reads mapped per sample	20,930,166	22,252,022	23,960,733	21,162,257	20,341,540	21,209,657

Table S4. List of auxin-related genes identified by RNA-Seq.

Type of DEG[a]	Gene ID	Log_2 fold change	P-value	Putative function
Up in e19[b]	LOC_Os01g02870	1.582	5.00E-05	auxin-induced protein 5NG4
Up in e19[b]	LOC_Os04g56850	0.851	0.06935	auxin response factor
Up in e19[b]	LOC_Os05g09480	0.991	5.00E-05	*OsIAA16* - Auxin-responsive Aux/IAA gene family member
Up in e19[b]	LOC_Os01g09450	1.301	5.00E-05	*OsIAA2* - Auxin-responsive Aux/IAA gene family member
Up in e19[b]	LOC_Os06g12610	1.01	5.00E-05	auxin efflux carrier component
Down in *lpa1*[c]	LOC_Os06g01966	-1.858	5.00E-05	auxin-induced protein 5NG4
Down in *lpa1*[c]	LOC_Os05g40330	-0.81	0.0105	auxin efflux carrier component

[a] indicates the type of differentially expressed gene (DEG).

[b] indicates upregulated genes in e19.

[c] indicates downregulated genes in *lpa1*

Table S5. Auxin sensitivity of mutants for lamina inclination

Genotypes	Auxin-sensitivity*	Comments (references)
lpa1	Hypersensitive	
LPA1-OX (e19)	Insensitive	
lpa1;d2	More sensitive than *lpa1*	*d2* accumulates 22-OHCR and 6-deoxoCT (Nakamura *et al*., 2009)
lpa1;d61	Similar to *lpa1*	
lpa1;bri-D	Similar to *lpa1*	
lpa1 treated with Brz	Less sensitive than *lpa1*	Brz inhibits C-22 hydroxylase (Nakamura *et al*., 2009)

* Relative sensitivity compared with wild type

Table S6. Relative sensitivity of *lpa1* and *lpa1,d61* to BR compounds for lamina inclination

BR compounds	Relative ratio		Comments
	lpa1 to WT*	*lpa1;d61* to *lpa1***	
22-OHCR (C-22-hydroxycampesterol)	4.1	0.6	
6-deoxo CT (cathasterone)	1.4	1.2	
6-deoxo TE (teasterone)	3.8	0.8	Substrates directly convert to active BR
6-deoxo 3DT (dehydroteasterone)	5.1	0.4	
6-deoxo TY (typhasterol)	1.9	0.8	
6-deoxo CS (castasterone)	7.8	0.4	

* Ratio of lamina angles in *lpa1* to those of wild type.

** Ratio of lamina angles in *lpa1; d61* to those of *lpa1*.

Supplementary Appendix (methods)

Appendix S1. Cloning, vector construction and transformation.

Full-length *LPA1* cDNA was isolated by RACE-PCR and RT-PCR. The 5’ and 3’ ends including the UTR (untranslated region) were cloned by RACE-PCR and the other part of the gene was amplified by RT-PCR. Primers used were as follows: 5’-ACCTGTCCGAC-TCCAGCAGCGTCCTCG-3’ for 5’ RACE-PCR; 5’-GTACAACTTGTACGTTC-ACGTGTGTA-3’ for 3’ RACE-PCR; 5’-CGGCATCAAGAAGCACTTC-3’ and 5’-GGATGATGGTGATGATGCCGCA-3’ for RT-PCR. RACE-PCR cDNA was synthesized using a BD SMART™ RACE cDNA Amplification Kit (Clontech, USA). RT-PCR cDNA was prepared from RNAs of shoot apices using Superscript II reverse transcriptase (Invitrogen, USA). To detect RNA beyond the *Ds* insertion site, the following primers were used: LPA1 RT-F, 5’-GCTACGCCGTCCAGTCCGACTACA-3’; LPA1 RT-R, 5’-GGCGGATGATGGTGATGATGCC-3’. To detect actin transcripts, the following primers

were used: ACT1 RT-F, 5’-CAGCATGGTATCGTCAGCAA-3’; ACT1 RT-R, 5’-GCAATGCCAGGGAACATAGT -3’. To clone the GUS fusion cDNA, RNAs from *lpa1::Ds* seedlings were reverse-transcribed and amplified with the following primers: LPA1 F, 5’-TCGGCATCAAGAAGCACTTC-3 and GUS R, 5’-CCAGACGTTGCCCGCATAATTAC-3. The cDNAs of the spliced forms were cloned into pBSK (Strategen) and eight clones were randomly selected and sequenced by Sanger sequencing. To generate *LPA1* overexpressors, the constitutive *Ubiquitin* promoter was fused with full-length *LPA1* cDNA by cloning into binary vector pGA1611. The construct was transformed into *Agrobacterium* strain ‘LBA4404’, which was introduced into callus of the Dongjin cultivar, from which transgenic plants were generated. To obtain the repressor domain, the C-terminal sequence (75 bp including the stop codon; amino acid sequence, PRPPADADADADAGLDLELRLAFF*) containing a conserved EAR motif of a gene (LOC_Os02g01090) orthologous to Arabidopsis *SUPERMAN* (At3g23130) (Hiratsu *et al*., 2004) was cloned into a plasmid vector. *LPA1* cDNA, whose stop codon was deleted, was fused to the C-terminal sequence of the gene. The fusion LPA1::repressor was expressed under the control of the *LPA1* promoter (from -2.4 kb to the start codon), which was cloned by PCR using genomic DNA. The following primers were used to clone the region -2400 bp from ATG: LPA1P-F, GGATCCACTCTGAAGCCTGAAAGTCTG; LPA1P-R, GTCGACAACTGCAAGGAGCTTTTCTTC.

Appendix S2. RNA-Seq analysis.

RNA sequencing experiments were performed to obtain transcriptome profiles of the lpa1

mutant, *LPA1* overexpressor (e19), and WT siblings. Using an RNeasy Plant Mini Kit (Qiagen, http://www.qiagen.com/), total RNA was extracted from 1 cm-long leaf segments spanning the second lamina joints of one week old seedlings. The mRNA was used to produce a library of template molecules suitable for subsequent cluster generation using the reagents provided in the Illumina® TruSeq™ RNA Sample Preparation Kit v2. The libraries were quantified using qPCR according to the qPCR Quantification Protocol Guide (KAPA Library Quantification kits for Illumina Sequencing platforms) and qualified using a Caliper Labchip GX (Caliper Life Sciences, USA). On average, 22 million Illumina paired end reads 101 bp in length were generated per replicate (Supplementary Table S2). Before data analysis, quality checking was performed using FastQC (Andrews, 2010). Next, adapter sequences were trimmed using Trim galore, a wrapper tool around Cutadapt and FastQC, in order to consistently provide quality and adapter trimming to FastQ files (Andrews, 2010). Trimmed reads were aligned to the rice genome sequence with the latest annotation files from rice genome annotation version 7 (ftp://ftp.plantbiology.msu.edu/pub/data/Eukaryotic_Projects/o_sativa/annotation_dbs/pseudomolecules/version_7.0/all.dir/). On average, 97% of the paired end reads were aligned to the genome (Supplementary Table S2). Approximately 16,000 genes showed at least one read aligned to both the control and mutant or OX. In the tophat pipeline, cuffmerge and cuffdif modules were sequentially used to identify differentially expressed genes (DEGs) (Trapnell et al., 2012). From the initial DEG set, genes with fragments per kilobase of exon per million fragments mapped (FPKM) values of at least four and P-value > 0.05 were considered to be DEGs.

References

Andrews, S. 2010. FastQC: a quality control tool for high throughput sequence data. Available online at: http://www.bioinformatics.babraham.ac.uk/projects/fastqc

Trapnell C, Roberts A, Goff L, Pertea G, Kim D, Kelley DR, Pimentel H, Salzberg SL, Rinn JL, Pachter L. 2012. Differential gene and transcript expression analysis of RNA-seq experiments with TopHat and Cufflinks. *Nature Protocol* **7,** 562–78.
